# Antibacterial activity of synthetic 1,3‐bis(aryloxy)propan‐2‐amines against Gram‐positive bacteria

**DOI:** 10.1002/mbo3.814

**Published:** 2019-02-17

**Authors:** Mateus S. M. Serafim, Stefânia N. Lavorato, Thales Kronenberger, Yamara V. Sousa, Graziele P. Oliveira, Simone G. dos Santos, Erna G. Kroon, Vinícius G. Maltarollo, Ricardo J. Alves, Bruno E. F. Mota

**Affiliations:** ^1^ Departamento de Análises Clínicas e Toxicológicas Faculdade de Farmácia Universidade Federal de Minas Gerais (UFMG) Belo Horizonte Brazil; ^2^ Departamento de Produtos Farmacêuticos Faculdade de Farmácia Universidade Federal de Minas Gerais (UFMG) Belo Horizonte Brazil; ^3^ Department of Internal Medicine VIII University Hospital Tübingen Tübingen Germany; ^4^ Departamento de Microbiologia Instituto de Ciências Biológicas Universidade Federal de Minas Gerais (UFMG) Belo Horizonte Brazil; ^5^ Present address: Centro das Ciências Biológicas e da Saúde Universidade Federal do Oeste da Bahia (UFOB) Rua Professor José Seabra de Lemos 316. CEP 47808‐021. Barreiras Bahia Brazil

**Keywords:** 3‐bis(aryloxy)propan‐2‐amines, antibacterial, gram‐positive bacteria, MRSA, synthetic 1, target prediction

## Abstract

Synthetic 1,3‐bis(aryloxy)propan‐2‐amines have been shown in previous studies to possess several biological activities, such as antifungal and antiprotozoal. In the present study, we describe the antibacterial activity of new synthetic 1,3‐bis(aryloxy)propan‐2‐amines against Gram‐positive pathogens (*Streptococcus pyogenes*,* Enterococcus faecalis* and *Staphylococcus aureus*) including Methicillin–resistant *S. aureus* strains. Our compounds showed minimal inhibitory concentrations (MIC) in the range of 2.5–10 μg/ml (5.99–28.58 μM), against different bacterial strains. The minimal bactericidal concentrations found were similar to MIC, suggesting a bactericidal mechanism of action of these compounds. Furthermore, possible molecular targets were suggested by chemical similarity search followed by docking approaches. Our compounds are similar to known ligands targeting the cell division protein *FtsZ*, Quinolone resistance protein *norA* and the Enoyl‐[acyl‐carrier‐protein] reductase *FabI*. Taken together, our data show that synthetic 1,3‐bis(aryloxy)propan‐2‐amines are active against Gram‐positive bacteria, including multidrug–resistant strains and can be a promising lead in the development of new antibacterial compounds for the treatment of these infections.

## Graphical abstract

The present study shows that some 1,3‐Bis(aryloxy)propan‐2‐amines inhibited the growth of Gram‐positive bacteria in the low micromolar range. Furthermore, these compounds were also effective in inhibiting the growth of clinical strains of methicillin–resistant *Staphylococcus aureus* (MRSA).

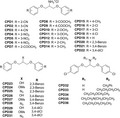

## 1 INTRODUCTION

Infections with antibiotic–resistant bacteria are a persistent problem to Public Health worldwide. Only in the US, at least 2 million people become infected with bacteria that harbor some type of resistance to commercially available antibiotics, of whom 23,000 die each year, ultimately estimating in $20 billion the increased health care costs (CDC, 2013). This scenario has become even worse if we consider that in the past 40 years only two classes of narrow–spectrum antibiotics (daptomicin and linezolid) were developed (Clatworthy, Pierson, & Hung, 2007). The scarcity of new therapeutic options against antibiotic–resistant strains has led to the return of older drugs previously disregarded due to its significant toxicity, such as colistin (Li et al., 2006). However, resistance mechanisms continue to emerge even for these drugs leading to the appearance of virtually untreatable infections (Malhotra‐Kumar et al., 2016).

Among the infections with resistant bacteria, one can high light the group of pathogens known as ESKAPE (*Enterococcus faecium*,* Staphylococcus aureus*,* Klebsiella pneumoniae*,* Acinetobacter baumannii*,* Pseudomonas aeruginosa*, and *Enterobacter* species). These infections are associated with longer periods of hospitalization, increases in hospital costs, higher use of antimicrobial drugs and higher mortality rates. The number of deaths caused by infection with methicillin–resistant *S. aureus* (MRSA) strains, for instance, surpassed the number of deaths from HIV/AIDS and tuberculosis combined in the US (Boucher et al., 2009).

The main strategy to overcome the problem of bacterial resistance is the development of new antibacterial agents. Regarding this strategy, the synthesis of new compounds and modification of the existing ones is promising and can extend the options of new drugs with a broader spectrum of activity, lower toxicity and/or reduced sensitivity to resistance mechanisms (Silver, 2011). This approach has resulted in the introduction of some new antibacterial agents for clinical use, such as retapamulin, a compound derived from pleuromutilin, and some of the classical modifications of penicillins, the aminopenicillins (Gao et al., 2017; Lobanovska & Pilla, 2017).

The 1,3‐Bis(aryloxy)propan‐2‐amines are a class of compounds synthesized by the amination of 1,3‐diaryloxypropyl toluenesulfonate, whose biological potential has not yet been extensively studied. We recently reported the trypanocidal (Lavorato, Sales Júnior, Murta, Romanha, & Alves, 2015) and leishmanicidal (Lavorato et al., 2017) activities of several compounds of this class, but their antibacterial action remains to be further studied.

**Figure 1:**
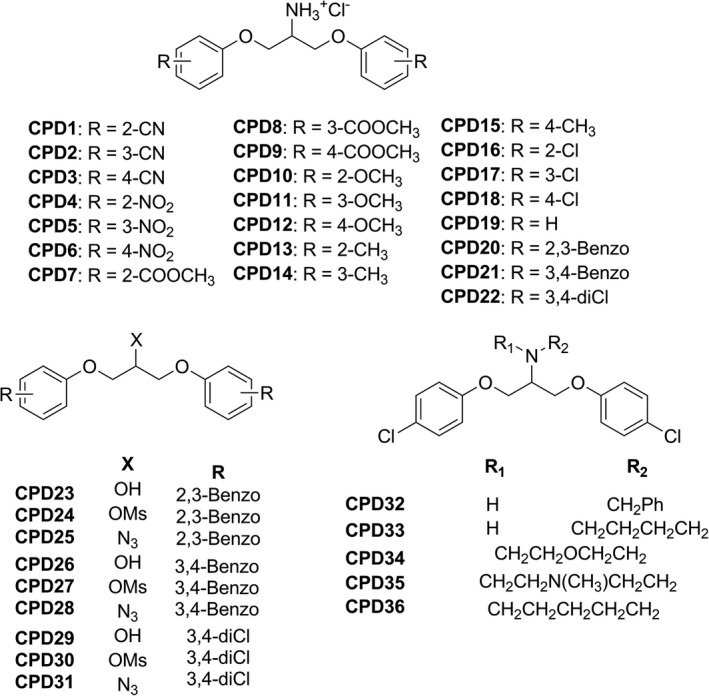
Compounds screened for antibacterial activity in the present study

In the present work we have evaluated the antibacterial activity of a series of 1,3‐bis(aryloxy)propan‐2‐amines, several synthetic intermediates and *N*–substituted amines (Figure 1).

## 2 MATERIALS AND METHODS

### 2.1 Chemistry

Compounds CPD1–CPD21, CPD23–CPD28 and CPD37 were synthesized and characterized by their IR, ^1^H and ^13^C NMR spectra and melting points as previously described (Lavorato et al., 2017). Compounds CPD22, CPD29–CPD36, CPD38 and CPD39 were synthesized and fully characterized as described in Appendix.

### 2.2 Cell lines

Vero cells (ATCC number CCL‐81) were maintained in Minimal Essential Medium (MEM; Cultilab, Brazil), while BSC‐40 cells (ATCC number CRL‐2761) were maintained in Dulbecco's Modified Essential Medium (DMEM; Cultilab, Brazil). Both media were supplemented with 5% fetal bovine serum (Cultilab, Brazil), 200 U/ml of penicillin, 100 μg/ml of streptomycin and 2.5 μg/ml of amphotericin B. The bacteria strains used were *Escherichia coli* (ATCC nº 35218), *K. pneumoniae* (ATCC nº 13883), *P. aeruginosa* (ATCC nº 27853), *E. faecalis* (ATCC nº 29212), *S. aureus* (ATCC n° 29213), *Streptococcus pyogenes* (ATCC nº 19615), MRSA (ATCC nº 43300) and five clinical strains of MRSA (*mecA* positives) (Gomes et al., 2015).

### 2.3 Minimal inhibitory concentration determination

The antibacterial activity of the compounds was evaluated using the broth microdilution method in 96‐well microplates according to the Clinical and Laboratory Standards Institute protocol (CLSI, 2017). First, synthetic compounds were diluted in Mueller Hinton broth (MHB; Oxoid, Thermo Scientific, UK) to concentrations ranging from 20 to 2.5 μg/ml. The same volume of a bacterial suspension containing 10^5^ CFU/ml was added to each of the previous solutions, resulting in final compound concentrations from 10 to 1.25 μg/ml. After incubation at 35°C for 24 hr, the plates were inspected visually for inhibition of bacterial growth. In each plate was included a viability control (bacterial suspension only), an inhibitory control (MHB containing five times the minimal inhibitory concentration (MIC) of penicillin G for Gram‐positive and Gentamicin for Gram–negative bacteria, or a serial dilution of vancomycin ranging from 16 to 2 μg/ml or 5.52 to 0.69 μM for MRSA strains) and a sterility control (medium only). All conditions were tested in triplicate and the results shown are representative of three independent assays.

### 2.4 Minimal bactericidal concentration determination

To evaluate the minimal bactericidal concentration (MBC) of tested compounds, the content of wells that showed no visual growth in the previous experiments, plus the well containing the viability control were plated in Mueller Hinton agar plates. After incubation at 35°C for 24 hr, the colonies were counted and the percentage of inhibition was calculated. MBC is defined as the lowest compound's concentration that inhibits at least 99.9% of the bacterial cell count compared to nontreated viability control (Clinical and Laboratory Standards Institute, 1999).

### 2.5 Cytotoxicity to mammalian cells

The cytotoxicity of active compounds to mammalian cells was assessed using the MTT reduction assay (Mosmann, 1983). Vero and BSC‐40 cells were seeded in 96‐well plates (8 × 10^4^ cells per well) and incubated at 37°C and 5% CO_2_ atmosphere. After 24 hr of incubation, 200 μL of fresh medium containing a serial dilution of compounds (10–1.25 μg/ml) were added to the plates. After 48 hr of incubation in the same conditions, 100 μL of MTT solution in MEM or DMEM (5 mg/ml) was added to each well and incubated for 3 hr at 37°C and 5% CO_2_ atmosphere. The medium was removed and 100 μL of DMSO was used to solubilize formazan crystals. Absorbance at 570 nm of each well was read using a spectrophotometer (VersaMax, Molecular Devices). The cytotoxic concentration of 50% (CC_50_) is defined as the lowest concentration of a specific compound that reduces by 50% the viability of cultured cells.

### 2.6 Putative molecular target identification by 3D chemical similarity and interaction profiling by molecular docking

First, the lowest energy conformations of tested compounds showing antibacterial activity were obtained by conformational analysis performed on OMEGA 2.5.1.4 software (Hawkins, Skillman, Warren, Ellingson, & Stahl, 2010). Then, the database of compounds with known effects over *S. aureus*,* S. pyogenes* and *E. faecalis* proliferation was retrieved from ChEMBL v23 (Bento et al., 2014). The three obtained databases were filtered to remove entries without experimental activity determined, inactive compounds and mixtures of compounds. For all compounds, the structures had their protonation states calculated according to pH = 7.4 using fixpka software implemented on QUACPAC 1.7.0.2 (OpenEye Scientific Software, 2016) and, then, the lowest energy conformers were generated using OMEGA.

Chemical similarity queries were created for each active compound by considering common chemical features (rings, H‐bond donors and acceptors, ions and hydrophobes) and the overall compound shape using the program ROCS 3.2.1.4 (Hawkins, Skillman, & Nicholls, 2007). ROCS software was used to identify the most similar compounds from the database against our queries. ROCS can overlay the library of conformers against a query composed of the shape and colors (representing chemical properties) derived from a compound. The output conformers were ranked according to their similarity with the query using a Tanimoto–combo coefficient (TC, a linear sum of Tanimoto coefficient for molecular shape and colors) and the compounds were considered for further analysis when TC > 1, representing at least 50% of chemical similarity (Rush, Grant, Mosyak, & Nicholls, 2005). Within this chemically similar dataset, compounds with experimental activity against molecular targets were identified and used in docking studies. Those targets were retrieved from the Protein Data Bank (PDB) or constructed using homology modeling.

Identified proteins were prepared by adding the adjusting protonation states of amino acids and fixing missing side–chain atoms (PrepWiz, Maestro v2017.4). Molecular docking was performed around the cocrystallized ligand of the different protein using the default settings of the Glide program (Glide v7.7, Maestro v2017.4) in extraprecision mode, with at least five poses selected for visual inspection (Friesner et al., 2006). The amino acid residues were considered rigid and structural water molecules were kept during calculation. The employed docking protocol was evaluated with redocking experiments. Our target prediction protocol was based on the previously published methodology (Vallone et al., 2018).

### 2.7 Homology modeling

Homology model of the *S. aureus* NorA (uniport accession number P0A0J7) was inferred using the *E. coli* homolog (PDB code: 4ZP0, resolution: 2.0 Å, sequence similarity: 77.3%) as a template. 3D model of the *Sa*NorA domain was generated using the online server HHPred (Söding, Biegert, & Lupas, 2005) for template identification and alignment followed by Modeller 9v19 (Eswar et al., 2006) for the model construction. The quality of the final structure was accessed by MolProbity (Davis, Murray, Richardson, & Richardson, 2004) showing three residues out of the Ramachandran allowed region, which was then fixed by the protein preparation step prior to docking.

## 3 RESULTS

**Figure 2:**
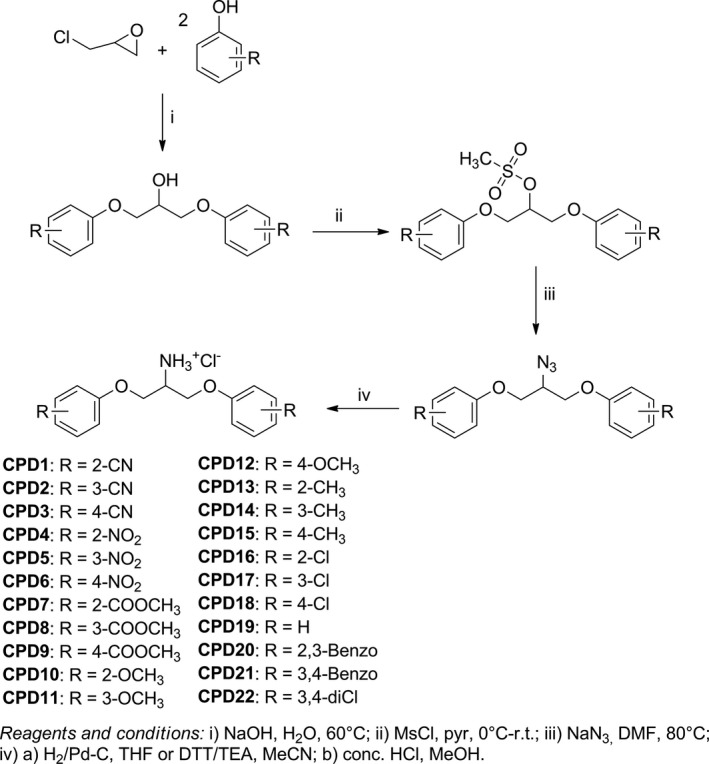
Synthesis of 1,3‐bis(aryloxy)propan‐2‐amines

To investigate the antibacterial potential of 1,3‐bis(aryloxy)propan‐2‐amines, 22 compounds of this class, variations in the nature and position of the substituents on the aromatic ring, were evaluated against Gram‐positive and Gram–negative bacteria. These compounds, named as CPD1–CPD22, were synthesized in four steps (Figure 2), as previously described by Lavorato et al. (2017).

### 3.1 Initial screening for antibacterial activity

Among the compounds initially tested, four—CPD18, CPD20, CPD21 and CPD22—presented antibacterial activity at the concentration of 10 μg/ml. Among the six bacterial species tested (*Escherichia coli*,* K. pneumoniae*,* P. aeruginosa*,* E. faecalis*,* S. aureus* and *S. pyogenes*), the activity was observed only against Gram‐positive bacteria. CPD20 and CPD22 inhibited the growth of all Gram‐positive bacteria tested (*E. faecalis*,* S. aureus* and *S. pyogenes*), while CPD18 and CPD21 showed activity against *S. aureus* and *S. pyogenes*.

### 3.2 Minimal inhibitory and MBC determination

Compounds that showed antibacterial activity in the initial screening were submitted to MIC determination by broth microdilution method. Among the four active compounds in the initial screening, CPD20 showed the best results, with MIC values of 2.5 μg/ml (6.58 μM) against *S. pyogenes* and *S. aureus* and 5 μg/ml (13.16 μM) against *E. faecalis*. CPD22 showed an MIC value of 2.5 μg/ml (5.99 μM) against *S. pyogenes* and 5 μg/ml (11.97 μM) against *S. aureus* and *E. faecalis*. On the other hand, both compounds CPD18 and CPD21 showed an MIC of 10 μg/ml (28.68 and 26.32 μM, respectively) against *S. aureus* and *S. pyogenes*.

To evaluate the MBC, the content of each well that showed no visual growth in the previous experiments were plated on MH agar and colony counts were compared to counts obtained from viability controls. Some of the MBC were equivalent to its MIC values suggesting a bactericidal activity of the tested compounds. CPD 21 showed equivalence between MBC and MIC values, against *S. aureus* and *S. pyogenes*. CPD20 also showed equivalence against *S. pyogenes* and CPD22 against *S. aureus*. CPD18 was an exception to this scenario, in which the MBC values were not equivalent to its MIC values against *S. aureus* and *S. pyogenes*. The results of quantitative antibacterial assays are summarized in Table 1.

**Table 1:** Minimal inhibitory concentration (MIC) and minimal bactericidal concentration (MBC) range (μM) for compounds that showed antibacterial activity in the initial screening

Compounds	MIC			MBC (MBC/MIC)		
	*Staphylococcus aureus* ATCC 29212	*Streptococcus pyogenes* ATCC 19615	*Enterococcus faecalis* ATCC 29213	*S. aureus* ATCC 29212	*S. pyogenes* ATCC 19615	*E. faecalis* ATCC 29213
CPD18	28.68	28.68	—	>28.68 (>1.0)	>28.68 (>1.0)	—
CPD20	6.58	6.58	13.16	26.32 (4.0)	6.58 (1.0)	26.32 (2.0)
CPD21	26.32	26.32	—	26.32 (1.0)	26.32 (1.0)	—
CPD22	11.97	5.99	11.97	11.97 (1.0)	23.95 (4.0)	>23.95 (>2.0)
Penicillin G	0.06	0.24	5.98	ND	ND	ND

*Note*.

Values presented are representative of at least three independent experiments.

ND: not determined.

### 3.3 Antibacterial activity against MRSA strains

In order to evaluate the efficacy of compounds against antibiotic resistant strains, we performed a broth microdilution method using MRSA. Corroborating the findings above, compound CPD20 showed MIC values of 2.5 μg/ml (6.58 μM) against all MRSA strains, being the most promising among all compounds tested. Values of MIC ranged from 2.5 to 5 μg/ml (5.99–11.97 μM) for CPD22 and from 5 to 10 μg/ml (13.16–26.32 μM) for CPD21. Compound CPD18 showed activity against four out of six MRSA strains, with MIC values of 10 μg/ml (28.68 μM). The MBC values were equivalent to its MIC values corroborating the hypothesis of a bactericidal activity of the tested compounds. CPD21 showed equivalence between MBC and MIC values against all MRSA strains. CPD20 showed the same values of MBC and MIC against MRSA strains nº 5749, 5912, 6100 and 6613 and CPD22 against MRSA 43300, 5749, 5912 and 6613. For compound CPD18, no bactericidal activity was observed for the concentrations tested. The results of antibacterial assays against MRSA strains are summarized in Table 2.

**Table 2:** Minimal inhibitory concentration (MIC) and minimal bactericidal concentration (MBC) range (μM) of the compounds against methicillin–resistant *Staphylococcus aureus* strains

Compounds	ATCC 43300	Strain 5749	Strain 5912	Strain 6100	Strain 6154	Strain 6613
MIC						
CPD18	—	28.68	28.68	—	28.68	28.68
CPD20	6.58	6.58	6.58	6.58	6.58	6.58
CPD21	—	26.32	13.16	26.32	26.32	26.32
CPD22	11.97	5.99	5.99	5.99	5.99	11.97
Vancomycin	1.38	1.38	1.38	1.38	1.38	1.38
MBC						
CPD18	—	>28.68	>28.68	—	>28.68	>28.68
CPD20	13.16	6.58	6.58	6.58	26.32	6.58
CPD21	—	26.32	13.16	26.32	26.32	26.32
CPD22	11.97	5.99	5.99	23.95	>23.95	11.97
Vancomycin	ND	ND	ND	ND	ND	ND

*Note*.

Values presented are representative of at least three independent experiments.

ND: not determined.

### 3.4 Changes in chemical group in R position abolish the antibacterial activity of tested compounds

**Figure 3:**
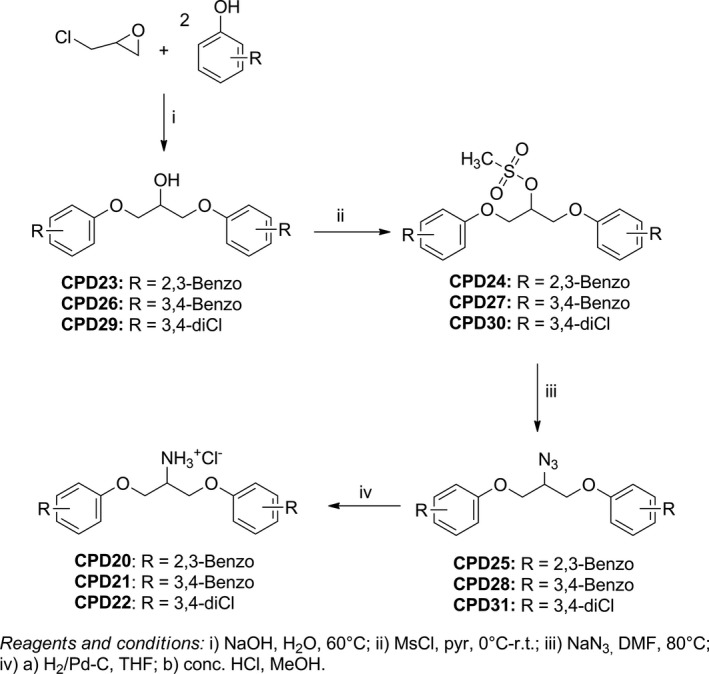
Synthesis of compounds CPD23–CPD31, synthetic intermediates of amines CPD20–CPD22

To investigate the importance of the amino group to antibacterial activity, compounds CPD23–CPD31, synthetic intermediates of the most active amines CPD20, CPD21 and CPD22, were selected for biological testing. As shown in Figure 3, they were obtained in one, two or three steps according to the substituent in C‐2.

**Figure 4:**
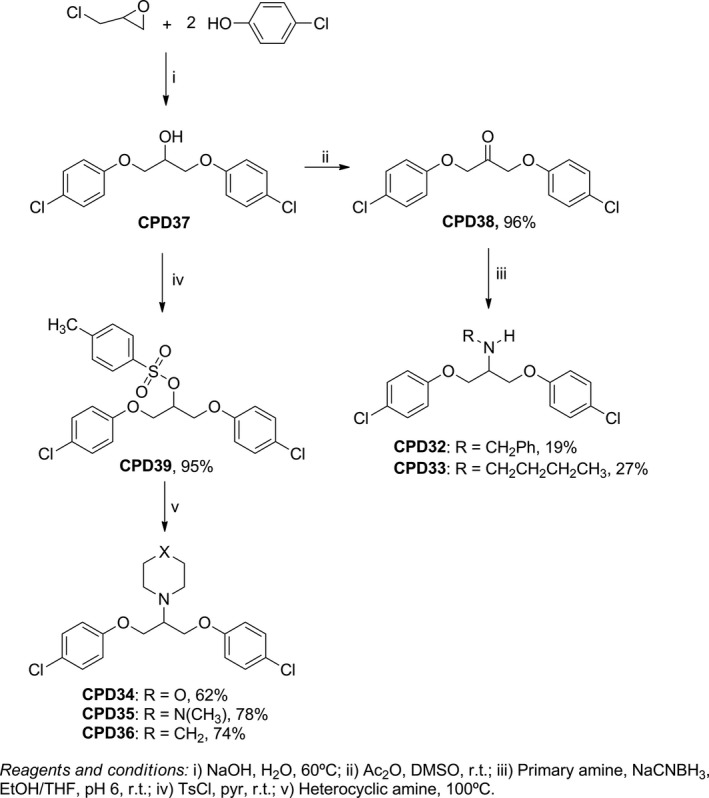
Synthesis of *N*‐substituted 1,3‐bis(aryloxy)propan‐2‐amines and yields of each synthetic step

In this second screening, we also prepared a series of secondary and tertiary amines for evaluation. As shown in Figure 4, compounds CPD38 and CPD39 were used as precursors to synthesize *N*‐substituted amines CPD32–CPD36 and both compounds were obtained from alcohol CPD37. CPD37 was obtained as previously described by Lavorato et al. (2017). The ketone CPD38 was obtained from CPD37 by Albright–Goldman oxidation using DMSO and acetic anhydride (Fritsche, Elfringhoff, Fabian, & Lehr, 2008), while the tosylate CPD39 was prepared by reacting CPD37 with *p*‐toluenesulfonyl chloride in dry pyridine (King & Bigelow, 1952). The secondary amines CPD32 and CPD33 were obtained by reductive amination reaction of CPD38 with benzylamine or butylamine, respectively, in the presence of NaCNBH_3_ as reducing agent (Borch, Bernstein, & Durst, 1971). The nucleophilic substitution reaction between CPD39 and the heterocyclic amines morpholine, *N*‐methylpiperazine and piperidine under heating at 100°C resulted in the tertiary amines CPD34, CPD35 and CPD36, respectively (Yuxiu, Guiqin, & Guangren, 2000).

None of these compounds presented antibacterial activity, with no complete inhibition of bacterial growth in all concentrations tested (up to 10 μg/ml, data not shown).

### 3.5 Cytotoxicity concentration (CC_50_) of active compounds in mammalian cells

We also evaluated the cytotoxicity in Vero and BSC‐40 cell lines of the active compounds using the colorimetric MTT assay. Compounds CPD18 and CPD22 showed higher values of CC_50_ for Vero (5.99 ± 0.09 μg/ml or 17.18 ± 0.26 μM and 5.47 ± 1.89 μg/ml or 13.1 ± 4.53 μM, respectively) and CPD18 and CPD21 for BSC‐40 (5.73 ± 0.18 μg/ml or 16.43 ± 0.52 μM and 5.06 ± 1.13 μg/ml or 13.32 ± 2.97 μM, respectively). The compound CPD20 showed the lowest values of CC_50_ (3.79 ± 0.60 μg/ml or 9.98 ± 1.58 μM in Vero and 2.50 ± 0.75 μg/ml or 6.58 ± 1.97 μM for BSC‐40 cells). In conjunction, CPD22 showed a higher Selective Index (SI) for both cell lines (2.19 in Vero and 1.77 for BSC‐40). The results of cytotoxicity assays are summarized in Table 3.

**Table 3:** Cytotoxic concentration of 50% (CC_50_) and selectivity index (SI) of the active compounds in Vero and BSC‐40 cells

Cell line	Compounds	CC_50_ ± *SD*	SI^a^								
			*Enterococcus faecalis*	*Staphylococcus aureus*	*Streptococcus pyogenes*	MRSA ATCC 43300	Strain 6613	Strain 5912	Strain 5749	Strain 6154	Strain 6100
Vero	*CPD18*	17.18 ± 0.26	—	0.6	0.6	—	0.6	0.6	0.6	0.6	—
	*CPD20*	9.98 ± 1.58	0.76	1.52	1.52	1.52	1.52	1.52	1.52	1.52	1.52
	*CPD21*	11.27 ± 2.58	—	0.43	0.43	—	0.43	0.86	0.43	0.86	0.43
	*CPD22*	13.1 ± 4.53	1.09	1.09	2.19	1.09	1.09	2.19	2.19	2.19	2.19
BSC‐40	*CPD18*	16.43 ± 0.52	—	0.57	0.57	—	0.57	0.57	0.57	0.57	—
	*CPD20*	6.58 ± 1.97	0.5	1.0	1.0	0.25	0.25	0.25	0.25	0.5	0.25
	*CPD21*	13.32 ± 2.97	—	0.51	0.51	—	0.51	1.01	0.51	0.51	0.51
	*CPD22*	10.59 ± 3.16	0.88	0.88	1.77	0.88	0.88	1.77	1.77	1.77	1.77

*Notes*.

Values presented are representative of at least three independent experiments.

MRSA: methicillin–resistant *S. aureus*.

a The SI is calculated by dividing the CC_50_ with the minimal inhibitory concentration.

### 3.6 The putative molecular targets of CDP20–22 and binding mode proposal

In order to identify the putative molecular target for the active compounds, we apply a ligand–based similarity approach combined with inverse docking using compounds CPD20, CPD21 and CPD22 as templates, since they presented stronger antibacterial activity in previous assays. Ligand–based similarity searches for each active compound were performed against a database of compounds with known effects against *S. aureus*,* S. pyogenes* and *E. faecalis*, resulting in 214, 15 and 30 unique compounds with at least 50% similarity against our hits respectively. Solely, *S. aureus* screening hits had an annotation for specific molecular targets, while the other two resulted in compounds with activity against whole cells or unchecked data (data not shown). The putative molecular targets for our hits in *S. aureus* with know 3D‐coordinates are namely the *ftsZ*, sortase and *FabI*, while *norA* has a known *E. coli* homolog (PDB code: 4ZP0, with 77.3% similarity) and underwent homology modeling (Table 4). The similarity search results suggest that CPD22 could have more than one molecular target.

**Table 4:** List of the putative molecular target of CPD20‐21‐22

Compound Chembl‐ID	Tanimoto combo			Related target (accession number)	Reference PMID	PDB
	CPD20	CPD21	CPD22			
CHEMBL499196	—	—	1.01	Cell division protein *FtsZ* P0A031	19064318	5XDV
CHEMBL461447	—	—	1.00		19064318	
CHEMBL1097797	—	—	1.02		20426423	
CHEMBL3098795	—	—	1.07		24287381	
CHEMBL3098796	—	—	1.05		24287381	
CHEMBL3417347	—	1.05	1.22	Quinolone resistance protein *norA* P0A0J7	25817769	Homology model
CHEMBL372191	1.01	—	—	Sortase Q9S446	1615474, 19269184	1QWZ
CHEMBL3623431	—	—	1.00	Enoyl‐[acyl‐carrier‐protein] reductase [NADPH] *FabI* Q9RMI3	26343826	4FS3

*Note*.

For each compound screened and hit combination the similarity index represented by the Tanimoto combo is presented. Protein accession number refers to the amino–acid sequence code at UniProt database and the Protein Data Bank (PDB) codes refer to the tridimensional structure deposited in the PDB.

Docking experiments were initially validated by redocking of the original ligand of each PDB file within their own active site. *Sa*NorA had the ligand of its homologous structure cross‐docked, both for defining the putative binding site, but also for verifying conserved interaction with important residues such as Asp34. Poses derivated from the redocking procedure had their heavy‐atom root mean squared deviation (RMSD) values calculated against the original cocrystallized conformation and its docking pose (Table A1).

**Figure 5:**
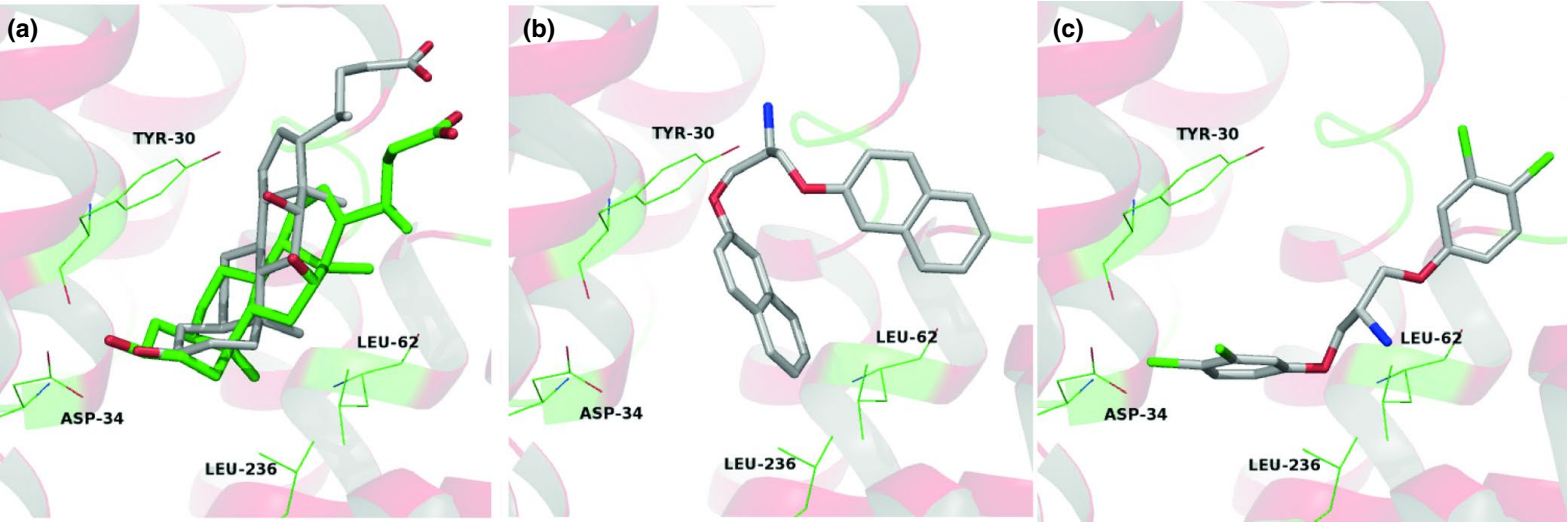
Cross‐docking of deoxycholate from *Ec*NorA (Protein Data Bank code: 4ZP0) ligand within the conserved binding cavity (a). Putative binding mode of CPD21 (b) and CPD22 (c) into the binding site of *Sa*NorA

*The Sa*NorA homology model shares high structural similarity with *E. coli*, however, cross‐docking performed moderately when compared to our redocking results (RMSD 1.20 Å, Figure 5a). CPD21 and CPD22 proposed interaction mode within the *Sa*NorA active site (Figure 5b,c) shares hydrophobic interactions mainly with Leu62 and Leu236, but not limited to, with also a large number of hydrophobic side‐chains surrounding both ring systems. Thai and collaborators by a comprehensive computational workflow have shown that *Sa*NorA has a conserved large binding site within the channel offering more opportunities for binding sites than the one exploited here in this study (Thai et al., 2015).

**Figure 6:**
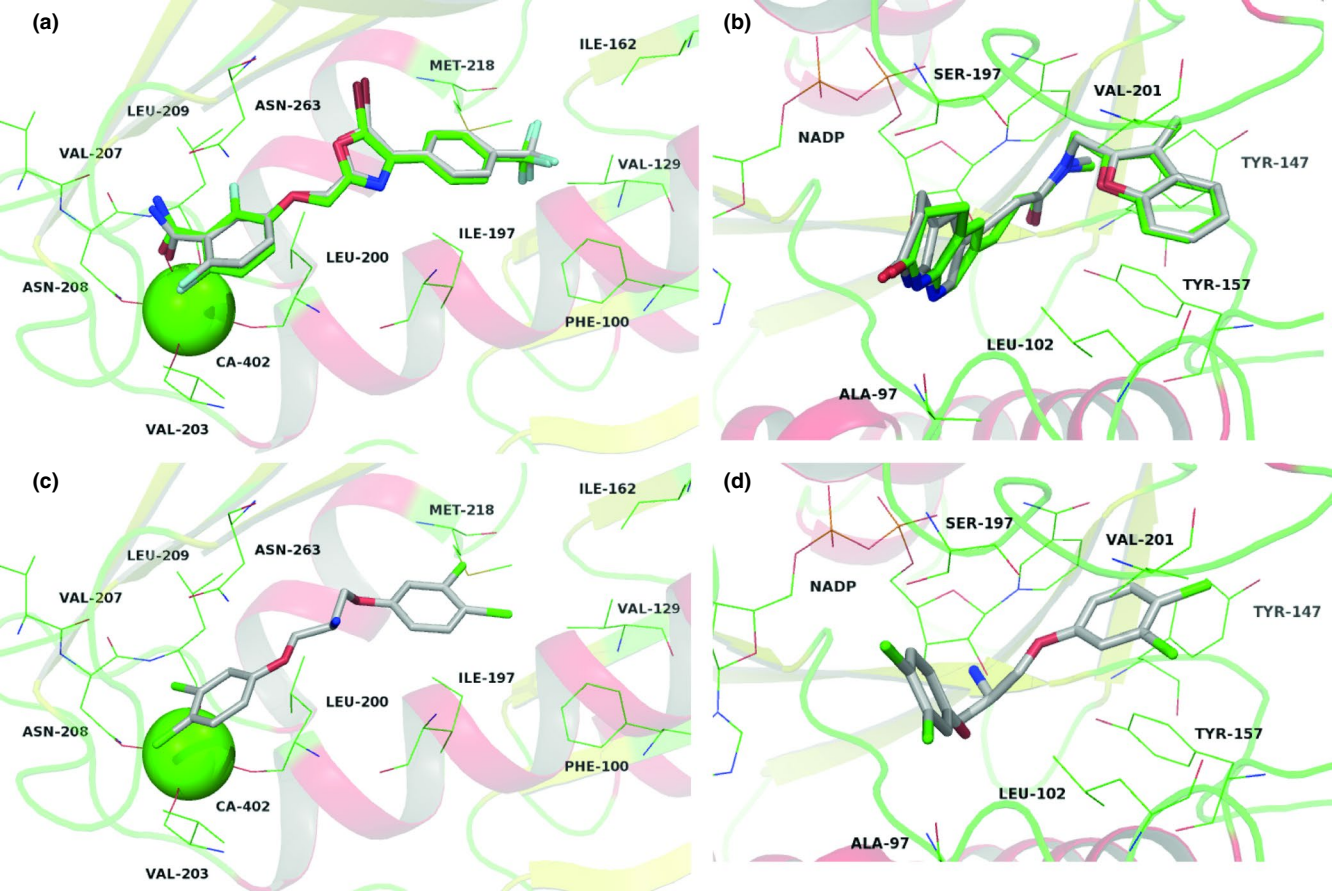
Redocking of original ligands TXA6101 from *Sa*FszT (a, Protein Data Bank [PDB] code: 5XDV) and AFN‐1252 into *Sa*FabI (b, PDB: 4FS3) into their respective described active site. Suggested binding mode by docking of CPD22 in the binding site of‐of FszT (c) and FabI (d)

CPD22 is suggested to have more than one target among the three–hit compounds, with two additional putative molecular targets, besides the *Sa*NorA: namely FabI and FstZ. Comparison between the redocked and cocrystallized conformations of TXA6101, within the FszT (PDB 5XDV, Figure 6a), and AFN‐1252, within FabI (PDB 4FS3, Figure 6b), showed very low conformational differences, validating the docking method.

FszT inhibition relies on a set of hydrogen bond interactions between the side‐chain of Asn163 and the main–chain atoms of Val207 and Leu209 with TXA6101 amide‐moyet but also has some significant apolar contacts with Val310, Ile311, Met225, Ile362, Met219 and Ile197. CPD22 ring shares most of these hydrophobic interactions, despite lacking the hydrogen interaction network (Figure 6c). Previous attempts on the application of virtual screening towards *Sa*FszT highlighted the importance of this hydrophobic complementarity with the ring systems of proposed inhibitors (Vijayalakshmi, Nisha, & Rajalakshmi, 2014).

The CPD22 resemblance with AFN‐1252 goes beyond the two ring structure, extending towards the compound interactions, both presenting interaction at the Tyr157 pocket (Figure 6d, Mistry, Truong, Ghosh, Johnson, & Mehboob, 2016). However, CPD22 does not have the typical Ala97 interaction previously described in the literature as an important chemical feature for FabI inhibition (Kronenberger et al., 2017; Mistry et al., 2016), but have a chlorine atom oriented at H‐bond region.

**Figure 7:**
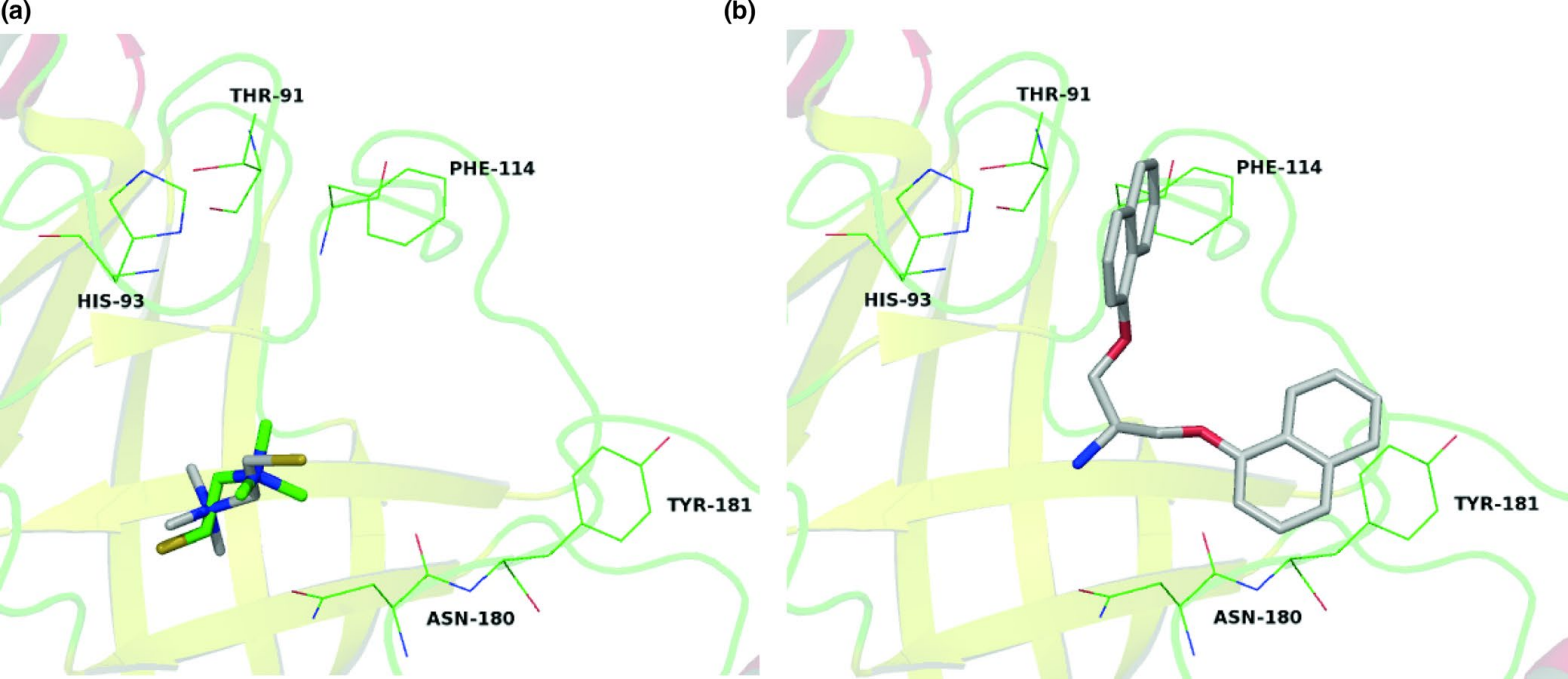
Redocking of original ligands 2‐(trimethylammonium)ethyl thiol from *Sa*Sortase (a, Protein Data Bank code: 1QWZ) and docking of CPD20 within the active site (b)

Lastly, the cysteine transpeptidase Sortase has been proposed as a putative molecular target for the CPD20. Sortase commonly binds to flexible ligands such as signaling peptides but can also be covalently inhibited by small compound fragments. Redocking in the PDB structure 1QWZ revealed moderate capacity of prediction for this target (Table A1 and Figure 7a), 1QWZ has a large binding site when compared to other sortase structures (Jacobitz et al., 2014). The two double–ring systems of CPD20 were positioned by docking near the aromatic residues Phe114 and Tyr181, however, no pi‐pi interactions could be established (Figure 7b).

Structural studies of the *Sa*Sortase B complexed with the substrate have shown a substrate–stabilized oxyanion hole involving Arg233 and Glu224 residues, which could accommodate the substrate (Jacobitz et al., 2014). Additionally, they also reported the close proximity of the ligands towards Tyr181, which could have a role in stabilizing the active conformation.

## 4 DISCUSSION

The compounds tested in the present study belong to the chemical class of 1,3‐bisaryloxypropan‐2‐amines, which have shown several biological activities in the literature and have easy access by synthesis (Heerding et al., 2003; Yuxiu et al., 2000). Our results showed that four out of 36 compounds presented antibacterial activity against the Gram‐positive bacteria tested (*E. faecalis*,* S. aureus* and *S. pyogenes*). The MIC values found in this study were in the low micromolar range, varying from 2.5 to 10 μg/ml (5.99–28.58 μM). The MIC values showed in our study are equal or even higher in comparison to the antimicrobials in clinical use against *S. aureus* and *E. faecalis* strains. For example, one can cite aminoglycosides such as amikacin (1–4 and 64–256 μg/ml for *S. aureus* and *E. faecalis*, respectively) and kanamycin (1–4 and 16–64 μg/ml), some beta‐lactams such as carbenicillin (2–8 and 16–64 μg/ml), piperacillin (1–4 μg/ml to both bacteria), methicillin (>16 μg/ml to *E. faecalis*) and ceftazidime (4–16 μg/ml to *S. aureus*), in addition to another important options as linezolid (1–4 μg/ml to both bacteria), chloramphenicol (2–16 and 4–16 μg/ml) and even vancomycin (0.5–2 and 1–4 μg/ml) (CLSI, 2017).

Other studies in the literature regarding the antibacterial activity of synthetic compounds have shown similar results, for example, Heerding et al. (2003) reported that an asymmetric diaryloxipropanamine showed antibacterial activity against *S. aureus*,* E. faecalis* and *S. pneumoniae* strains with MIC values of 16, 32 and 2 μg/ml, respectively (Heerding et al., 2003). Other synthetic compounds derived from pleuromutilin also had similarresults, showing MIC values between 0.06 and 32 μg/ml for *S. aureus* and between 1.0 and 32 μg/ml for *E. faecalis* (Gao et al., 2017).

Moreover, the pleuromutilin derivates mentioned above also had comparable efficacy against MRSA strains, with MIC values ranging from 0.015 to over 32 μg/ml. Other studies have also shown antibacterial activity of synthetic molecules against MRSA, such as synthetic biphenylthiazoles, which presented MIC values ranging from 0.39 to 25 μg/ml against three different MRSA strains (Hagras et al., 2017). In fact, equivalent MIC values were obtained in our study when compounds were tested against MRSA strains ranging from 2.5 to 10 μg/ml (5.99 to 28.58 μM). Those values are also comparable to some antimicrobials used in the clinic for MRSA, such as daptomycin (0.5 μg/ml), vancomycin (2 μg/ml), oxacillin (16 μg/ml) and gentamicin (128 μg/ml) (Baltch, Ritz, Bopp, Michelsen, & Smith, 2007).

Regarding the MBC assay, results ranging from 2.5 to 10 μg/ml (5.99 to 26.32 μM) were in the same range of MIC values suggesting the bactericidal mode of action of these compounds. This characteristic is desirable for an antibacterial drug since it is often associated with the capability of inhibiting and preventing bacterial dissemination (Alder & Eisenstein, 2004). For example, synthetic biphenylthiazoles have presented MBC values ranging from 8 to 32 μg/ml against *S. aureus* strains.

In the initial screening for antibacterial activity at the concentration of 10 μg/ml, only four compounds showed Gram‐positive antibacterial activity. The *p*‐chloro‐substituted CPD18 was the only monosubstituted aromatic compound, with an MIC of 10 μg/ml (28.68 μM) against *S. aureus*,* S. pyogenes* and several MRSA strains. The introduction of a second chloro‐substituent at position 3 on aromatic rings, as observed in CPD22, a 3,4‐dichloro–substituted aromatic compound, potentiate the activity against Gram‐positive bacteria, including *E. faecalis,* reducing the MIC values to 2.5 to 5 μg/ml (5.99–11.97 μM). Although not essential, the dissubstitution pattern seems to be important for antibacterial activity, since three of the four active compounds, namely CPD20, CPD21 and CPD22, have aromatic rings substituted at two positions. The 1‐naphthyloxy derivative (CPD20) presents an antibacterial activity against all Gram‐positive bacteria evaluated, including MRSA strains with MIC values ranging from 2.5 to 5 μg/ml (6.58–13.16 μM), similar to the values determined for CPD22. The 2‐naphthyloxy derivative (CPD21), with MIC values of 10 μg/ml (26.32 μM), demonstrated antibacterial activity against *S. pyogenes*,* S. aureus* and MRSA strains similar to compound CPD18, however its activity against *E. faecalis* was not observed.

The synthetic intermediates of active compounds CPD20, CPD21 and CPD22 were also evaluated in order to get some insights about the role of an amino group in the aliphatic chain to the antibacterial activity of these compounds. The substitution of the amino group by a hydroxyl, a mesyl or an azide group leads to loss of activity (CPD23–31), indicating that this group is essential for the antibacterial activity. Since we recognize the importance of an amino group placed in the aliphatic chain, we also verified if the substitution of the amino group would interfere with their activity, synthetizing and evaluating secondary and tertiary amines derived from CPD18. The results indicated a loss of activity when the amino group is substituted (CPD32–36), suggesting that these compounds need to be a primary amine to promote antibacterial effects.

Among the evaluated primary amines, CPD18, CPD20, CPD21 and CPD22 are the ones with the highest calculated partition coefficient (ClogP) values (Table A2). The ClogP is a measure of lipophilic character of a compound which increases as the lipophilicity of the compound increases. Thus, this indicates that the highest lipophilicity of these three compounds contributes to a better Gram‐positive antibacterial activity. This relationship is similar to that observed to the antibacterial activity of cephalosporins and penicillins against *S. aureus* (Biagi, Guerra, Barbaro, & Gamba, 1970). This trend could indicate that the activity of primary amines to Gram‐positive bacteria could be related to the bacterial membrane permeability in which substituent as chloro, dichloro and naphtyl could improve.

Although in most cases the highest lipophilic character may be a limiting factor to the use of these compounds in oral or intravenous formulas, they could favor its topical use, like in ointments and emulsions due to the direct absorption of lipophilic substances. This route of administration may be beneficial for the treatment of surgical site infections, decubitus ulcers, mainly caused by species of *Enterococcus*,* S. *and *Staphylococcus*, especially MRSA strains.

The cytotoxic concentration of 50% (CC_50_) of active compounds in mammalian cells was generally also in the low micromolar range, corroborating the results obtained by Lavorato et al. (2017). Hence, the selectivity index (SI) values obtained ranged from 0.25 to 2.19. These low SI values can be improved by changing critical chemical groups in the molecule, which can reduce its toxicity or enhance its activity.

Here, experimental validation demonstrated the ability of CPD20, CPD21 and CPD22 to interfere with the growth of *S. aureus*,* S. pyogenes* and *E. faecalis*. However, the molecular target of these drug candidates remains undetermined. In order to identify putative protein targets, we have employed a combination of chemical similarity search with inverse docking approaches. The three–dimensional chemical similarity between our hits and compounds with known activity against the organisms of interest was used to select a set of compounds with known biological targets. The prediction of the binding mode suggests that the compounds can interact with same pockets/regions of known cocrystallized inhibitors, which indicate the possibility of CPD22 to be a multi‐target antibacterial. Furthermore, for the suggested targets, both 3D chemical similarity and parallels in terms the protein–ligand interactions between our compounds with known inhibitors supports this binding mode and encourages further in vitro testing. For instance, the dichloro–benzene groups of CPD22 interacting with the *FabI* hydrophobic pocket (Figure 6d).

Taken together, our data show that some compounds belong to the class of symmetric 1,3‐bis(aryloxy)propan‐2‐amine tested in the present study showed a relevant antibacterial activity against important Gram‐positive pathogens (including antibiotic–resistant strains), with minimal inhibitory and bactericidal concentration in the low micromolar range. As a perspective, we intend to investigate the activity of this class of amines against other clinically relevant resistant bacteria, such as *E. faecium*, vancomycin–resistant enterococci and glycopeptide–intermediate *S. aureus*. Through an in silico approach, we identified three putative molecular targets for these compounds and we hope that these data may contribute, in the long‐term, to lead these compounds for further optimization towards selectivity, aiming to treat bacterial infections, including those caused by resistant Gram‐positive bacteria.

## CONFLICT OF INTEREST

The authors declare that there is no conflict of interest.

## AUTHORS CONTRIBUTION

M.S.M.S. performed the experiments, analyzed the results and wrote the manuscritpt; S.N.L. synthetized the compounds and wrote the manuscritpt; T.K. performed the in silico experiments and wrote the manuscritpt; Y.V.S. performed the experiments; G.P.O. performed the experiments to evaluate the cytotoxicity of the compounds; S.G.S. provided the clinical MRSA strains; E.G.K. analyzed the results and wrote the manuscritpt; V.G.M. performed the in silico experiments, analyzed the results and wrote the manuscritpt; R.J.A. synthetized the compounds, analyzed the results and wrote the manuscritpt; B.E.F.M. coordinated the Project, design experiments, analyzed the results and wrote the manuscritpt.

## ETHICS STATEMENT

None required.

## Data Availability

The data that support the findings of this study are available from the corresponding author upon request.

## APPENDIX 1

### SYNTHESIS AND CHARACTERIZATION DATA OF COMPOUNDS CPD22, CPD29–CPD36, CPD38 AND CPD39

All reagents were obtained from commercial suppliers and used without further purification, unless stated otherwise. IR spectra were obtained using a Spectrum One, Perkin‐Elmer ATR system. Melting points were determined on Microquímica MQAPF 301 apparatus. ^1^H and ^13^C NMR spectra were obtained on a Bruker Avance DPX‐200 spectrometer and proton and carbon chemical shifts (δ) are reported with respect to Tetramethylsilane (TMS). Column chromatography was carried out on silica gel 60 0.063–0.200 mm/70–230 mesh Merck. Pyridine was dried over KOH pellets. DMSO was dried over 3Å molecular sieves. Anhydrous DMF was obtained from commercial supplier (Sigma‐Aldrich).

#### 1,3‐Bis(3,4‐dichlorophenoxy)propan‐2‐ol (CPD29)

Epichlorohydrin (1 ml; 12.8 mmol) was added dropwise to a stirred solution of 3,4‐dichlorophenol (38 mmol) and sodium hydroxide (38 mmol) in water (30 ml). The reaction mixture was stirred under heating (60°C) for 14 hr. After this time, the reaction mixture was cooled to room temperature and the product was isolated as a white solid using vacuum filtration in 88% yield; mp: 103.4–105.7°C. IR (ATR) *v*_max_ 3,527, 2,948, 1,589, 1,566, 1,478, 1,465, 1,455, 1,227, 1,064, 1,121/cm; ^1^H NMR (CDCl_3_, 200 MHz): δ = 7.33 (2H, d, *J *=* *9.0 Hz, H‐5′), 7.03 (2H, d, *J *=* *2.6 Hz, H‐2′), 6.78 (2H, dd, *J *=* *9.0 Hz, 2.6 Hz, H‐6′), 4.37 (1H, s, H‐2), 4.11 (4H, d, *J *=* *4.8 Hz, H‐1), 2.59 (1H, s, OH); ^13^C NMR (CDCl_3_, 50 MHz): δ = 157.5 (C, C‐1′), 133.2 (C, C‐3′), 131.0 (CH, C‐5′), 125.0 (C, C‐4′), 116.7 (CH, C‐2′), 114.7 (CH, C‐6′), 69.3 (CH_2_, C‐1), 68.6 (CH, C‐2).

#### 1,3‐Bis(3,4‐dichlorophenoxy)propan‐2‐yl methanesulfonate (CPD30)

To a stirred and ice‐cooled solution of CPD29 (1.57 mmol) in pyridine (3 ml), methanesulfonyl chloride (6.3 mmol; 0.49 ml) was added dropwise. Crushed ice was added to the flask after 4 hr of reaction, followed by the addition of concentrated HCl until pH 1. The product was extracted with dichloromethane (3 × 30 ml). The organic layers were combined, washed with water (5 × 50 ml), dried over anhydrous Na_2_SO_4_, filtrated and concentrated under reduced pressure. A white solid was obtained in 83% yield; mp: 83.7–84.4°C. IR (ATR) *v*_max_ 3,042, 2,940, 2,883, 1,593, 1,566, 1,478, 1,452, 1,345, 1,231, 1,176, 1,057/cm; ^1^H NMR (CDCl_3_, 200 MHz): δ = 7.35 (2H, d, *J *=* *8.8 Hz, H‐5′), 7.03 (2H, d, *J *=* *3.0 Hz, H‐2′), 6.78 (2H, dd, *J *=* *8.8 Hz, 3.0 Hz, H‐6′), 5.21 (1H, qn, *J *=* *5.2 Hz, H‐2), 4.29 (4H, d, *J *=* *5.2 Hz, H‐1), 3.15 (3H, s, OSO_2_CH_3_); ^13^C NMR (CDCl_3_, 50 MHz): δ = 156.9 (C, C‐1′), 133.4 (C, C‐3′), 131.2 (CH, C‐5′), 125.5 (C, C‐4′), 116.8 (CH, C‐2′), 114.6 (CH, C‐6′), 77.1 (CH, C‐2), 67.4 (CH_2_, C‐1).

#### 1,3‐Bis(3,4‐dichlorophenoxy)propan‐2‐yl azide (CPD31)

Sodium azide (10.9 mmol) was added to a stirred solution of CPD30 (1.09 mmol) in DMF (3 ml) at 80°C. Crushed ice was added to the flask after 24 hr of reaction. The product was extracted with dichloromethane (3 × 30 ml) and the organic layer was washed with water (5 × 50 ml). The organic layers were combined, dried over anhydrous Na_2_SO_4_, filtrated and concentrated under reduced pressure. A white solid was obtained after recrystallization from isopropyl alcohol in 67% yield; mp: 95.6–96.3°C. IR (ATR) *v*_max_ 2,940, 2,882, 2,144, 2,094, 1,590, 1,567, 1,478, 1,454, 1,231, 1,023/cm; ^1^H NMR (CDCl_3_, 200 MHz): δ = 7.34 (2H, d, *J *=* *8.8 Hz, H‐5′), 7.03 (2H, d, *J *=* *2.8 Hz, H‐2′), 6.79 (2H, ddd, *J *=* *8.8 Hz, 2.8 Hz, 0.4 Hz, H‐6′), 4.20‐4.11 (5H, m, H‐1, H‐2); ^13^C NMR (CDCl_3_, 50 MHz): δ = 157.2 (C, C‐1′), 133.3 (C, C‐3′), 131.1 (CH, C‐5′), 125.3 (C, C‐4′), 116.7 (CH, C‐2′), 114.7 (CH, C‐6′), 68.0 (CH_2_, C‐1), 59.3 (CH, C‐2).

#### 1,3‐Bis(3,4‐dichlorophenoxy)propan‐2‐aminium chloride (CPD22)

10% Palladium on activated carbon (20 mg) was added to a solution of CPD31 (0.47 mmol) in Tetrahydrofuran (THF) (10 ml). The reaction was kept under stirring and hydrogen atmosphere for 4 hr. Then, the catalyst was removed using filtration and the filtrate was concentrated. The residue was reconstituted in methanol and concentrated hydrochloric acid was added dropwise until a slight precipitate is formed. The solvent was evaporated to give a white solid in 41% yield; mp: 224.1–225.6°C. IR (ATR) *v*_max_ 2,882, 1,589, 1,569, 1,511, 1,478, 1,460, 1,231, 1,049/cm; ^1^H NMR (DMSO‐*d*_*6*_, 200 MHz): δ = 8.79 (3H, s, NH_3_^+^Cl^−^), 7.56 (2H, d, *J *=* *8.8 Hz, H‐5′), 7.32 (2H, d, *J *=* *2.8 Hz, H‐2′), 7.05 (2H, dd, *J *=* *8.8 Hz, 2.8 Hz, H‐6′), 3.90 (1H, dd, *J *=* *5.2 Hz, *J *=* *4.6 Hz, H‐2), 4.39 (2H, dd, *J *=* *10.5 Hz, *J *=* *4.6 Hz, H‐1a), 4.31 (2H, dd, *J *=* *10.5 Hz, *J *=* *5.2 Hz, H‐1b); ^13^C NMR (DMSO‐*d*_*6*_, 50 MHz): δ = 157.5 (C, C‐1′), 131.6 (C, C‐3′), 131.1 (CH, C‐5′), 123.3 (C, C‐4′), 116.9 (CH, C‐2′), 115.8 (CH, C‐6′), 65.8 (CH_2_, C‐1), 49.0 (CH, C‐2).

#### Synthesis of 1,3‐bis(4‐chlorophenoxy)propan‐2‐one (CPD38)

Acetic anhydride (17.67 mmol) was added to anhydrous dimethyl sulfoxide (9 ml; 127 mmol) and the solution was kept under nitrogen atmosphere for 10 min. This solution was then added dropwise to a solution of CPD37 (3.19 mmol). The reaction mixture was stirred for 24 hr. Thereafter, a 1:1 mixture of saturated solutions of NaHCO_3_ and NaCl was added to the flask and a white solid was isolated using vacuum filtration in 96% yield; mp: 84.4–87.9°C. IR (ATR) *v*_max_ 3,098, 3,063, 3,045, 2,958, 2,924, 2,890, 1,731, 1,594, 1,585, 1,487, 1,243, 1,223, 1,062, 815/cm; ^1^H NMR (acetone‐*d*_*6*_, 200 MHz): δ = 7.31 (4H, d, *J *=* *9.0 Hz, H‐3′, H‐5′), 7.00 (4H, d, *J *=* *9.0 Hz, H‐2′, H‐6′), 5.05 (4H, s, H‐1); ^13^C NMR (acetone‐*d*_*6*_, 50 MHz): δ = 201.6 (C, C‐2), 158.0 (C, C‐1′), 130.2 (CH, C‐3′, C‐5′), 126.7 (C, C‐4′), 117.2 (CH, C‐2′, C‐6′), 72.1 (CH_2_, C‐1).

#### Synthesis of 1,3‐bis(4‐chlorophenoxy)propan‐2‐yl 4‐methylbenzenesulfonate (CPD39)

4‐Toluenesulfonyl chloride (5.76 mmol) was added to a solution of CPD37 (1.92 mmol) in pyridine (3 ml), under stirring in ice‐bath. After 2 hr, crushed ice was added to the flask and then concentrated hydrochloric acid was added until pH 1, leading to the product precipitation. A white solid was isolated by vacuum filtration in 95% yield; mp: 145.3–147.2°C. IR (ATR) *v*_max_ 3,094, 2,930, 1,598, 1,581, 1,491, 1,458, 1,349, 1,171, 1,244, 1,041, 827/cm; ^1^H NMR (CDCl_3_, 200 MHz): δ = 7.80 (4H, d, *J *=* *8.0 Hz, H‐2″, H‐6″), 7.30 (4H, d, *J *=* *8.0 Hz, H‐3″, H‐5″), 7.20 (2H, d, *J *=* *8.8 Hz, H‐3′, H‐5′), 6.69 (2H, d, *J *=* *8.8 Hz, H‐2′, H‐6′), 5.01 (1H, t, *J *=* *5.0 Hz, H‐2), 4.21 (4H, d, *J *=* *5.0 Hz, H‐1), 2.44 (1H, s, OSO_2_PhCH_3_); ^13^C NMR (CDCl_3_, 50 MHz): δ = 156.6 (C, C‐1′), 145.3 (C, C‐1″), 130.0 (CH, C‐3″, C‐5″), 129.6 (C, CH, C‐3′, C‐5′, C‐4″), 128.3 (CH, C‐2″, C‐6″), 126.7 (CH, C‐4′), 116.0 (CH, C‐2′, C‐6′), 77.3 (CH, C‐2), 66.7 (CH_2_, C‐1), 21.9 (CH_3_, OSO_2_PhCH_3_).

#### General procedure 1 for synthesis of N‐substituted amines

To a solution of the appropriate primary amine in THF and absolute ethyl alcohol (30 ml, 1:1) was added a methanolic solution of hydrochloric acid 5 M until pH 6. Then, CPD38 and NaCNBH_3_ were added. The reaction was stirred under room temperature for 72 hr. The reaction was quenched with water (1 ml). The solvent was then removed under reduced pressure, and the residue was reconstituted in dichloromethane (30 ml) and washed with 0.5 M aqueous hydrochloric acid and water (3 × 30 ml). The organic layers were combined, dried over anhydrous Na_2_SO_4_, filtrated and concentrated under reduced pressure.

#### General procedure 2 for synthesis of N‐substituted amines

A mixture of CPD39 and the appropriated secondary amine was heated to 100°C for 24 hr without stirring. Then, ethyl acetate (30 ml) was added to the flask and the resulting mixture was transferred to a separatory funnel. This mixture was washed with water (3 × 50 ml). The organic layers were combined, dried over anhydrous Na_2_SO_4_, filtrated and concentrated under reduced pressure.

#### 4,4′‐((2‐(Benzyloxy)propane‐1,3‐diyl)bis(oxy))bis(chlorobenzene) (CPD32)

From benzylamine (3.6 mmol), CPD38 (0.6 mmol) and NaCNBH_3_ (1.2 mmol) and using the general procedure 1, a colorless oil was isolated using silica gel column chromatography (Hexanes/EtOAc 9:1 v/v) in 19% yield; IR (ATR) *v*_max_ 3,336, 3,063, 3,028, 2,925, 2,875, 2,849, 1,595, 1,581, 1,489, 1,462, 1,235, 1,028, 819/cm; ^1^H NMR (CDCl_3_, 200 MHz): δ = 7.37 (5H, s, H‐5, H‐6, H‐7, H‐8, H‐9), 7.25 (4H, d, *J *=* *6.0 Hz, H‐3′, H‐5′), 6.83 (4H, d, *J *=* *6.0 Hz, H‐2′, H‐6′), 4.10 (4H, s, H‐1), 3.98 (2H, s, H‐3), 3.40 (1H, s, H‐2), 2.04 (1H, s, NH); ^13^C NMR (CDCl_3_, 50 MHz): δ = 157.6 (C, C‐1′), 140.3 (C, C‐4), 129.6 (CH, C‐3′, C‐5′), 128.7 (CH, C‐6, C‐8), 128.3 (CH, C‐5, C‐9), 127.4 (CH, C‐7), 126.2 (C, C‐4′), 116.1 (CH, C‐2′, C‐6′), 68.1 (CH_2_, C‐1), 55.9 (CH_2_, C‐1), 68.6 (CH, C‐2).

#### N‐(1,3‐Bis(4‐chlorophenoxy)propan‐2‐yl)butan‐1‐amine (CPD33)

From butylamine (1.92 mmol), CPD38 (0.32 mmol) and NaCNBH_3_ (0.64 mmol) and using the general procedure 1, a colorless oil was isolated using silica gel column chromatography (Hexanes/EtOAc 9:1 v/v) in 27% yield; IR (ATR) *v*_max_ 2,956, 2,927, 2,871, 1,595, 1,581, 1,489, 1,463, 1,235, 1,031, 1,005, 819/cm; ^1^H NMR (CDCl_3_, 200 MHz): δ = 7.22 (4H, d, *J *=* *9.0 Hz, H‐3′, H‐5′), 6.84 (4H, d, *J *=* *9.0 Hz, H‐2′, H‐6′), 4.06 (4H, d, *J *=* *5.6 Hz, H‐1), 3.32 (1H, qn, *J *=* *5.6 Hz, H‐2), 2.74 (2H, t, *J *=* *6.6 Hz, H‐3), 1.96 (1H, s, NH), 1.59‐1.26 (4H, m, H‐4, H‐5), 0.92 (3H, t, *J *=* *7.2 Hz, H‐6); ^13^C NMR (CDCl_3_, 50 MHz): δ = 157.5 (C, C‐1′), 129.6 (C, C‐3′, C‐5′), 126.1 (C, C‐4′), 116.0 (CH, C‐4′, C‐6′), 67.9 (CH_2_, C‐1), 56.7 (CH, C‐2), 47.7 (CH_2_, C‐3), 32.6 (CH_2_, C‐4), 20.6 (CH_2_, C‐5), 14.1 (CH_3_, C‐6).

#### 4‐(1,3‐Bis(4‐chlorophenoxy)propan‐2‐yl)morpholine (CPD34)

CPD34 was obtained from morpholine (2.1 mmol) and CPD39 (0.21 mmol) using the general procedure 2. The crude residue was triturated with absolute ethyl alcohol and the solid impurities were filtered using vacuum filtration. The filtrate was evaporated under reduced pressure to give a brown solid in 62% yield; mp: 62.6–64.8°C; IR (ATR) *v*_max_ 2,955, 2,855, 1,595, 1,581, 1,491, 1,468, 1,238, 1,023, 822/cm; ^1^H NMR (acetone‐*d*_*6*_, 200 MHz): δ = 7.30 (4H, d, *J *=* *9.0 Hz, H‐3′, H‐5′), 7.01 (4H, d, *J *=* *9.0 Hz, H‐2′, H‐6′), 4.31 (2H, dd, *J *=* *10.2 Hz, 5.5 Hz, H‐1a), 4.25 (2H, dd, *J *=* *10.2 Hz, 5.5 Hz, H‐1b), 3.27 (1H, qn, *J *=* *5.5 Hz, H‐2), 3.60 (4H, t, *J *=* *4.4 Hz, H‐3), 2.81 (4H, t, *J *=* *4.4 Hz, H‐4); ^13^C NMR (acetone‐*d*_*6*_, 50 MHz): δ = 158.7 (C, C‐1′), 130.2 (CH, C‐3′, C‐5′), 126.1 (C, C‐4′), 117.2 (CH, C‐2′, C‐6′), 68.2 (CH_2_, C‐1), 67.3 (CH_2_, C‐4), 63.4 (CH, C‐2), 51.7 (CH_2_, C‐3).

#### 1‐(1,3‐Bis(4‐chlorophenoxy)propan‐2‐yl)‐4‐methylpiperazine (CPD35)

From N‐methylpiperazine (3.2 mmol) and CPD38 (0.32 mmol) and using the general procedure 1, a colorless oil was isolated using silica gel column chromatography (dichloromethane/methanol 98:2 v/v) in 78% yield; IR (ATR) *v*_max_ 2,935, 2,878, 2,839, 1,595, 1,580, 1,489, 1,467, 1,455, 1,235, 1,027, 1,006, 820/cm; ^1^H NMR (acetone‐d_6_, 200 MHz): δ = 7.28 (4H, d, J = 8.8 Hz, H‐3′, H‐5′), 6.99 (4H, d, J = 8.8 Hz, H‐2′, H‐6′), 4.26 (2H, s, H‐1a), 4.23 (2H, s, H‐1b), 3.28 (1H, s, H‐2), 2.81 (4H, s, H‐3), 2.34 (4H, s, H‐4), 2.16 (3H, s, H‐5); ^13^C NMR (acetone‐d_6_, 50 MHz): δ = 158.7 (C, C‐1′), 130.2 (CH, C‐3′, C‐5′), 126.0 (C, C‐4′), 117.2 (CH, C‐2′, C‐6′), 67.4 (CH_2_, C‐1), 62.9 (CH, C‐2), 56.7 (CH_2_, C‐3), 50.8 (CH_2_, C‐4), 46.5 (CH_3_, C‐5).

#### 1‐(1,3‐Bis(4‐chlorophenoxy)propan‐2‐yl)piperidine (CPD36)

From piperidine (3.9 mmol) and CPD38 (0.39 mmol) and using the general procedure 1, a brown oil was isolated using silica gel column chromatography (dichloromethane 100%) in 74% yield; IR (ATR) *v*_max_ 2,933, 2,852, 2,808, 1,595, 1,580, 1,489, 1,467, 1,235, 1,031, 1,017, 819/cm; ^1^H NMR (CDCl_3_, 200 MHz): δ = 7.23 (4H, d, *J *=* *8.6 Hz, H‐3′, H‐5′), 6.86 (4H, d, *J *=* *8.6 Hz, H‐2′, H‐6′), 4.18 (4H, d, *J *=* *5.4 Hz, H‐1), 3.23 (1H, t, *J *=* *5.4 Hz, H‐2), 2.74 (4H, s, H‐3), 1.60 (4H, s, H‐4), 1.48 (2H, s, H‐5); ^13^C NMR (CDCl_3_, 50 MHz): δ = 157.6 (C, C‐1′), 129.5 (CH, C‐3′, C‐5′), 126.0 (C, C‐4′), 116.2 (CH, C‐2′, C‐6′), 66.8 (CH_2_, C‐1), 63.0 (CH, C‐2), 52.0 (CH_2_, C‐3), 26.9 (CH_2_, C‐4), 24.8 (CH_2_, C‐5).

**Table 5:** Redocking validation protocol (and cross‐docking for NorA) and docking pose scores (kcal/mol). RMSD values expressed in Ångström (Å) was calculated from the comparison between redocking results with the cocrystallized conformation and are shown between parenthesis after the calculated energy. Poses are ranked by their GlideScore XP with more negative values representing more energetically stable interactions. Protein structures are described by their Protein Data Bank (PDB) codes, where HM stands for Homology Model

CPD20	Sortase	CPD21	NorA	CPD22	FabI	FstZ	NorA
PDB code	1QWZ		HM	PDB code	4FS3	5XDV	HM
Redocking	−4.98 (1.76 Å)	Cross‐docking	−7.03 (1.20 Å)	Redocking	−9.74 (0.63 Å)	−11.59 (0.41 Å)	−7.03 (1.20 Å)
Pose 1	−3.25	Pose 1	−8.50	Pose 1	−9.53	−8.79	−3.66
Pose 2	−2.87	Pose 2	−7.96	Pose 2	−9.08	−8.13	−3.59
Pose 3	−2.66	Pose 3	−3.43	Pose 3	−8.83	−8.07	−1.86
Pose 4	−2.51			Pose 4	−7.46	−7.90	
Pose 5	−2.24			Pose 5	−7.32	−5.20	

**Table 6:** Calculated partition coefficient (ClogP) of compounds CPD1–CPD22

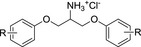

Compound	R	ClogP*
CPD1	2‐CN	1.95
CPD2	3‐CN	1.11
CPD3	4‐CN	1.95
CPD4	2‐NO_2_	1.67
CPD5	3‐NO_2_	2.55
CPD6	4‐NO_2_	2.27
CPD7	2‐COOCH_3_	2.30
CPD8	3‐COOCH_3_	2.38
CPD9	4‐COOCH_3_	2.75
CPD10	2‐OCH_3_	2.13
CPD11	3‐OCH_3_	2.07
CPD12	4‐OCH_3_	2.41
CPD13	2‐CH_3_	3.40
CPD14	3‐CH_3_	3.40
CPD15	4‐CH_3_	3.40
CPD16	2‐Cl	3.57
CPD17	3‐Cl	4.14
CPD18	4‐Cl	3.85
CPD19	H	2.48
CPD20	2,3‐Benzo	4.94
CPD21	3,4‐Benzo	4.94
CPD22	3,4‐diCl	5.26

* Calculated using ACD/ChemSketch software (acdlabs.com).
